# Leaf hydroalcoholic extract and oleoresin from *Copaifera multijuga* control *Toxoplasma gondii* infection in human trophoblast cells and placental explants from third-trimester pregnancy

**DOI:** 10.3389/fcimb.2023.1113896

**Published:** 2023-02-13

**Authors:** Aryani Felixa Fajardo Martínez, Samuel Cota Teixeira, Guilherme de Souza, Alessandra Monteiro Rosini, Joed Pires de Lima Júnior, Gabriel Nogueira Melo, Kelvin Orlando Espinoza Blandón, Angelica Oliveira Gomes, Sergio Ricardo Ambrósio, Rodrigo Cassio Sola Veneziani, Jairo Kenupp Bastos, Carlos Henrique Gomes Martins, Eloisa Amália Vieira Ferro, Bellisa Freitas Barbosa

**Affiliations:** ^1^ Laboratory of Immunophysiology of Reproduction, Institute of Biomedical Sciences, Federal University of Uberlândia, Uberlândia, Brazil; ^2^ Laboratory of Biology of Reproduction, Institute of Biomedical Sciences, Federal University of Uberlândia, Uberlândia, Brazil; ^3^ Institute of Natural and Biological Sciences, Federal University of Triângulo Mineiro, Uberaba, Brazil; ^4^ Nucleus of Research in Technological and Exact Sciences, University of Franca, Franca, Brazil; ^5^ School of Pharmaceutical Sciences of Ribeirão Preto, University of São Paulo, Ribeirão Preto, Brazil; ^6^ Laboratory of Antimicrobial Testing, Institute of Biomedical Sciences, Federal University of Uberlândia, Uberlândia, Brazil

**Keywords:** *Toxoplasma gondii*, congenital toxoplasmosis (CT), trophoblast, *Copaifera multijuga*, hydroalcoholic extract, oleoresin

## Abstract

The conventional treatment of congenital toxoplasmosis is mainly based on the combination of sulfadiazine and pyrimethamine. However, therapy with these drugs is associated with severe side effects and resistance, requiring the study of new therapeutic strategies. There are currently many studies with natural products, including *Copaifera* oleoresin, showing actions against some pathogens, as *Trypanosoma cruzi* and *Leishmania*. In the present study, we investigated the effects of the leaf hydroalcoholic extract and oleoresin from *Copaifera multijuga* against *Toxoplasma gondii* in human villous (BeWo) and extravillous (HTR8/SVneo) trophoblast cells, as well as in human villous explants from third-trimester pregnancy. For this purpose, both cells and villous explants were infected or not with *T. gondii*, treated with hydroalcoholic extract or oleoresin from *C. multijuga* and analyzed for toxicity, parasite proliferation, cytokine and ROS production. In parallel, both cells were infected by tachyzoites pretreated with hydroalcoholic extract or oleoresin, and adhesion, invasion and replication of the parasite were observed. Our results showed that the extract and oleoresin did not trigger toxicity in small concentrations and were able to reduce the *T. gondii* intracellular proliferation in cells previously infected. Also, the hydroalcoholic extract and oleoresin demonstrated an irreversible antiparasitic action in BeWo and HTR8/SVneo cells. Next, adhesion, invasion and replication of *T. gondii* were dampened when BeWo or HTR8/SVneo cells were infected with pretreated tachyzoites. Finally, infected and treated BeWo cells upregulated IL-6 and downmodulated IL-8, while HTR8/SVneo cells did not change significantly these cytokines when infected and treated. Finally, both the extract and oleoresin reduced the *T. gondii* proliferation in human explants, and no significant changes were observed in relation to cytokine production. Thus, compounds from *C. multijuga* presented different antiparasitic activities that were dependent on the experimental model, being the direct action on tachyzoites a common mechanism operating in both cells and villi. Considering all these parameters, the hydroalcoholic extract and oleoresin from *C. multijuga* can be a target for the establishment of new therapeutic strategy for congenital toxoplasmosis.

## Introduction

*Toxoplasma gondii* is an obligate intracellular protozoan parasite belonging to the Apicomplexa phylum, responsible for serious morbidities mainly related to newborns and immunocompromised people (Blader et al., 2015; Zhang et al., 2019). It is estimated that a third of the world population is infected with this microorganism, making it one of the most successful microorganisms (Montoya and Liesenfeld, 2004). Congenital toxoplasmosis is considered as one of the most serious forms of the disease, caused by the transplacental passage of *T. gondii* tachyzoites acquired during or just before pregnancy. It triggers serious implications for the fetal development and may go unreported into adolescence or adulthood (Kodjikian, 2010). Primary infection during pregnancy can lead to spontaneous abortion, stillbirth, premature delivery, malformations and neurological or ocular disorders in newborns (Carlier et al., 2012; Oz, 2014).

An immune response, preferentially T helper 1 (Th1), is induced when the parasites invade the host cells, requiring components of the innate and adaptive immune response (Carruthers, 2002). Initially, *T. gondii* is recognized by innate immune response cells, which stimulate interleukin (IL)-12 release by dendritic cells, macrophages and neutrophils, and induce interferon (IFN)-γ production by natural killer (NK) cells (Fitch et al., 1975). The production of these proinflammatory cytokines is associated with the activation of the adaptive immune response mediated by CD4^+^ and CD8^+^ T cells, which produce high levels of IFN-γ as well as other proinflammatory cytokines, besides triggering the release of various inflammatory mediators, such as nitric oxide (NO) by macrophages (Buzoni-Gatel et al., 2006; Miller et al., 2009). In addition to IFN-γ and IL-12, other proinflammatory cytokines are of great importance in controlling *T. gondii* infection, such as IL-6 and macrophage migration inhibitory factor (MIF) (Castro et al., 2013; Souza et al., 2021). Therefore, the production of these proinflammatory cytokines stands out as a robust and classic defense mechanism against *T. gondii* infection in the host. In parallel, IL-8 has been considered an important cytokine during *T. gondii* infection. This cytokine is a member of the CXC chemokine family and it is an important mediator during innate immune response to several pathogens (Dong and Zheng, 2015). Studies showed that *T. gondii* has the ability to attract IL-8 and this cytokine can recruit inflammatory cells to local infection, attracting neutrophil and immature dendritic cells and, consequently, promoting a dissemination of the infection by host organism (Sommerville et al., 2013). Then, IL-8 can be an important mediator to favor the establishment of the infection. In addition, anti-inflammatory cytokines such as IL-10 and transforming growth factor (TGF)-β are necessary to prevent an exacerbated immune response that could be detrimental to the host (Fowler et al., 1976). The immune response is not sufficient to clear the infection. In this sense, the establishment of new drugs are mandatory for infected pregnant women and congenitally infected children.

When maternal infection is present, but without evidence of fetal infection, the classical therapy is based in spiramycin, a macrolide antibiotic that prevents congenital transmission (Elsheikha, 2008; Tamaru et al., 2011; Peyron et al., 2017). In the case of confirmed fetal infection, the first treatment option is the combination of pyrimethamine and sulfadiazine (Villena et al., 1998; Doliwa et al., 2013). The coadministration of folinic acid becomes necessary to minimize the toxic effects of pyrimethamine (Montoya and Remington, 2008). It should be noted that sulfadiazine is associated with gastrointestinal disturbances, and patients often do not tolerate this chemotherapy (Montoya and Remington, 2008). Furthermore, approximately half of the patients treated with spiramycin retained *Toxoplasma* DNA in their blood for a long time (Habib, 2008). Therefore, finding active and less toxic drugs as new therapeutic strategies to prevent or treat congenital toxoplasmosis is absolutely necessary and encouraging. Our previous studies have shown alternative drugs or potential molecular targets with great effects against *T. gondii* infection (Costa et al., 2009; Souza et al., 2021; Teixeira et al., 2021). Thus, it is important to highlight the importance of studying alternative treatments for the control of *T. gondii* infection.

In this scenario, the use of natural products and their isolated molecules has emerged as an interesting tool for the discovery of new relevant substances with a great antiparasitic action against *T. gondii.* Huang et al. (2021) used five essential oleoresins (EOs) and evaluated their antiparasitic activity against *T. gondii* in fibroblast cells (HFF). EOs from *Eucalyptus globulus*, *Cupressus sempervirens*, *Citrus aurantifolia* and *Melaleuca alternifolia* did not show any effect in *T. gondii* infection. However, EO from *Pelargonium X. Asperum* (PaEO) inhibited the growth of *T. gondii* in a dose-dependent manner. Also, another study conducted by Sharif et al. (2019) evaluated the effect of the hydroalcoholic extract from the stem of *Tinospora crispa* (EETC) against *T. gondii* using Vero cells. The authors observed that EETC induced more than 70% and 80% reduction in invasion rate and parasite intracellular proliferation, respectively. In addition, Miranda et al. (2021) investigated the action of the hydroalcoholic extract of *Annona muricata* (EtOHAm) and its fractions in the control of *T. gondii*, showing that the HexAm, CH2Cl2Am and EtOAcAm fractions exerted anti-*Toxoplasma* effect *in vitro*; however, only EtOHAm was effective in improving survival and decreasing tissue parasitism in C57BL/6 mice.

*Copaifera* genus, also popularly known as “pau d’oleo”, belongs to the Fabaceae family (Leguminosae). The representative species of this genus are found mainly in the American and African continents (Veiga-Júnior and Pinto, 2002). *Copaifera* oleoresin is a natural substance composed of a non-volatile solid resinous part formed by diterpenic acids responsible for 55 to 60% of its constitution, which is diluted in another volatile part, the essential oleoresin composed of sesquiterpenes (Cascon and Gilbert, 2000). *Copaifera* oleoresin has sesquiterpenes, diterpenes and β-caryophyllene as its main compound, possessing healing and antiseptic properties, in addition to being considered as anti-inflammatory and antibiotic (Santos et al., 2008; Souza et al., 2011; Arruda et al., 2019). Several studies have demonstrated the antiparasitic action of the oleoresin and isolated components from *Copaifera* or other members from the Fabaceae family, especially with trypanocidal, leishmanicidal and antiplasmodial activities, through direct action on parasites and/or by modulating the immune response (Izumi et al., 2013; Tomani et al., 2021). Extracts and purified molecules of various species of the genera *Piper*, *Tanacetum*, *Porophyllum* and *Copaifera* exhibited interesting antitrypanosomal and antileishmanial activities. These natural compounds affected different structures in the parasites, suggesting that the mitochondria are the strategic target to induce parasite death (Lazarin-Bidóia et al., 2022).

However, few studies have addressed the action of these compounds against *T. gondii.* Recently, Teixeira et al. (2020) demonstrated the ability of four *Copaifera* oleoresins (*C. reticulata, C. duckei, C*. *paupera* and *C. pubiflora*) to impair *T. gondii* infection in human villous trophoblast cells (BeWo lineage) and human villous explants from third-trimester pregnancy. However, there are no studies about the possible effects of the hydroalcoholic extract and oleoresin from *C. multijuga* against *T. gondii* infection in maternal-fetal interface. *C. multijuga* is found in the Amazon and is now being studied extensively due to its various properties, the most expressive being its anti-inflammatory, anticancer and antimicrobial (antiseptic) properties (Pinheiro et al., 2017).

In this sense, the present study aims to demonstrate, for the first time, the anti-*T. gondii* effects of the leaf hydroalcoholic extract and oleoresin from *C. multijuga* in human trophoblast cells. As experimental models, we used BeWo and HTR8/SVneo cells, representatives of human villous and extravillous trophoblasts, respectively, as well as human villous explants as an experimental model of maternal-fetal interface. Our research group widely uses these cells and explants in studies about human maternal-fetal interface (Barbosa et al., 2014; Barbosa et al., 2015; Teixeira et al., 2020; Souza et al., 2021; Teixeira et al., 2021).

## Material and methods

### Cell culture

BeWo cells were commercially obtained from the American Type Culture Collection (ATCC, Manassas, VA, USA), while HTR8/SVneo cells were a gift from Dr. Estela Bevilacqua (University of São Paulo, São Paulo, SP, Brazil). Both cells were cultured in RPMI 1640 medium (Cultilab, Campinas, SP, Brazil) supplemented with 100 U/mL penicillin (Sigma Chemical Co., St. Louis, MO, USA), 100 μg/mL streptomycin (Sigma) and 10% fetal bovine serum (FBS) (Cultilab) at 37°C and 5% CO_2_.

### Human chorionic villous explant culture

Human third-trimester placentas (36 to 40 weeks of pregnancy), n = 4, were collected after elective cesarean deliveries at the Clinics Hospital of the Federal University of Uberlândia (HC-UFU), MG, Brazil. Placental tissues were collected based on exclusion criteria, as follows: pre-eclampsia, chronic hypertension, infectious diseases including toxoplasmosis, chorioamnionitis, chronic kidney disease, heart disease, connective tissue disease, pre-existing diabetes mellitus, gestational diabetes mellitus and other pathological manifestations. Briefly, placental tissues were washed in sterile PBS to remove excess blood, then aseptically dissected to remove endometrial tissue and fetal membranes up to 1 h after collection. Terminal chorionic villi containing five to seven free tips per explant were harvested and added to 96-well microplates (one villus per well) in 200 µL/well of fresh RPMI 1640 medium supplemented with 100 U/mL penicillin, 100 µg/mL streptomycin and 10% FBS for 24 h at 37°C in a humidified atmosphere containing CO_2_ (5%) (Silva et al., 2017).

### Parasite culture

*Toxoplasma gondii* tachyzoites (2F1 clone) constitutively expressing the β-galactosidase gene were maintained by serial passages in BeWo cells cultured in medium containing 2% FBS, 100 U/mL penicillin and 100 μg/mL streptomycin at 37°C and 5% CO_2_. 2F1 clone is derived from the highly virulent strain RH and was a gift from Dr. Vern Carruthers, School of Medicine from the University of Michigan (USA).

### Oleoresin and leaf hydroalcoholic extract from *C. multijuga*

The samples of oleoresin and leaf hydroalcoholic extract from *C. multijuga* were provided by Professor Carlos Henrique Gomes Martins from the Department of Microbiology (DEMIC), Institute of Biomedical Sciences (ICBIM), Federal University of Uberlândia (UFU), Uberlândia, MG, Brazil. Authorization to carry out scientific studies with plant species from the Brazilian biodiversity was requested from the Council for Authorization and Information on Biodiversity (SIBIO/ICMBio/MMA/BRAZIL) and Genetic Heritage Management (CGEN/MMA/BRAZIL). Authorizations to carry out research activities with these plants were issued under numbers 35143-1 and 010225/2014-5, respectively.

Authentic oleoresin was collected in the North region, in the state of Amazonas, in the city of Manacapuru, by Jonas J. M. da Silva by drilling the tree trunks using a 2” drill bit. The exudates removed were stored in glass bottles. All the plant material collected was identified by Silvane Tavares Rodrigues, a voucher specimen (NID 03/2013 and 62/2013) was deposited in the Herbarium of the Brazilian Agricultural Research Corporation (Embrapa Eastern Amazon), by direct comparison with authentic herbarium vouchers, of which a taxonomic identity certificate is available upon request.

Air dried leaves (40 °C for 48 h) of *C. multijuga* (200* g*) were grounded and then exhaustively extracted by maceration with 1.2 L of ethanol/H_2_O (7:3) at room temperature for 48 h to afford 50 g of *C. multijuga* hydroalcoholic extract of leaves after lyophilization (Pimenta et al., 2019).

### Cell viability after treatment with oleoresin or hydroalcoholic extract from *C. multijuga*

We verified the toxicity of the compounds selected in BeWo and HTR8/SVneo cells. For this purpose, we performed the MTT [(3-(4,5-dimethylthiazol-2-yl)-2,5-diphenyltertrazolin bromide)] colorimetric assay, following the protocol described by Mosmann (1983). BeWo (3×10^4^ cells/well/200 µL) and HTR8/SVneo (1.5×10^4^ cells/well/200 µL) cells were cultured in 96-well plates for 24 h in RPMI 1640 medium with 10% FBS at 37°C and 5% CO_2_. Next, cells were treated in serial dilutions with increasing concentrations of oleoresin or hydroalcoholic extract from *C. multijuga* in a medium with 10% FBS for 24 h. In parallel, both cells were treated with DMSO (0.8%), the percentage used to obtain higher dilution for both oleoresin and hydroalcoholic extract. As control, cells were not treated and received only medium. Then, the supernatants were removed and the cells incubated with 10 µL MTT (5 mg/mL) plus 90 µL of RPMI 10% FBS medium for 4 h at 37°C and 5% CO_2_. Formazan crystals resulting from cellular metabolism were solubilized by adding a solution containing 10% sodium dodecyl sulfate (SDS, Sigma) and 50% N,N-dimethyl formamide (Sigma) for 30 min (Mosmann, 1983). Optical densities were measured at 570 nm on a plate reader (Versa Max ELISA Microplate Reader, Molecular Devices, Sunnyvale, CA, USA). Data were expressed as the percentage of viable cells (cell viability %) in comparison to untreated cells (100% of cell viability). Three independent experiments were performed in eight replicates.

### *T. gondii* intracellular proliferation after treatment with oleoresin or hydroalcoholic extract from *C. multijuga*

We selected different concentrations of oleoresin and hydroalcoholic extract from *C. multijuga* after MTT assay and tested their effect on *T. gondii* growth. BeWo (3×10^4^ cells/well/200 µL) and HTR8/SVneo (1.5×10^4^ cells/well/200 µL) cells were cultured in 96-well plates for 24 h in RPMI 1640 medium with 10% FBS at 37°C and 5% CO_2_. Next, both cells were infected with *T. gondii* tachyzoites at a multiplicity of infection (MOI) of 3:1 (parasites per cell). After 3 h, the medium was discarded, extracellular parasites were retired by washing with medium, and the cells were treated with non-toxic concentrations of oleoresins or hydroalcoholic extracts from *C. multijuga* for additional 24 h at 37°C and 5% CO_2_ as follows: for BeWo cells, 4 to 32 µg/mL; for HTR8/SVneo, 4 to 16 µg/mL. Sulfadiazine and pyrimethamine (SDZ + PYR, 200 + 8 µg/mL for BeWo, and 100 + 4 µg/mL for HTR8/SVneo) was used as positive control (Silva et al., 2017). BeWo or HTR8/SVneo cells infected by *T. gondii* and non-treated were used as negative controls. After 24 h of treatment, the culture supernatants were collected and stored at -80°C for further measurement of cytokines. In parallel, *T gondii* intracellular proliferation was analyzed using a colorimetric β-galactosidase assay (Silva et al., 2017; Souza et al., 2021). We quantified *T. gondii* intracellular proliferation and calculated the number of tachyzoites in comparison with a standard curve containing free tachyzoites (1×10^6^ to 15,625×10^3^ parasites). Infected BeWo or HTR8/SVneo cells treated only with medium (negative control) represented uninhibited parasite growth. We calculated the means of untreated cells (medium) that corresponded to 100% of *T. gondii* proliferation. Then, all values about number of tachyzoites obtained from all treatments were compared to 100%, and data demonstrated in % of *T. gondii* proliferation. Three independent experiments were performed in eight replicates.

### Reversibility and reinfection assays

In order to evaluate the maintenance of the antiparasitic effects of oleoresins and hydroalcoholic extracts in *T. gondii* growth, we firstly performed the reversibility test (Teixeira et al., 2020). Briefly, BeWo (3×10^4^ cells/well/200 µL) or HTR8/SVneo (1.5×10^4^ cells/well/200 µL) cells were seeded in 96-well plates as above described. After 24 h in culture, cells were infected with *T. gondii* tachyzoites (3:1) for 3 h, washed to remove extracellular parasites and treated for two conditions as follows: (1) BeWo cells were treated with oleoresin or hydroalcoholic extract (both 16 or 32 µg/mL), HTR8/SVneo cells were treated with oleoresin or hydroalcoholic extract (both 8 or 16 µg/mL), or both cells received SDZ + PYR (positive control) or only medium (negative control), then parasite intracellular proliferation was performed after 24 h in culture conditions; (2) cells and parasites were cultured in the same conditions as previously described, but after 24 h of treatment, the cells were washed, the medium replaced by new RPMI 1640 medium without treatments, and the parasites were allowed to grow for additional 24 h. In both situations, we quantified the *T. gondii* proliferation using the β-galactosidase assay as mentioned above. Finally, we measured the percentage reversibility rate (treatment reversibility %) at 24 h after treatment removal compared to the untreated group (considered as 100% reversibility) and the corresponding treatment condition at 24 h of treatment (baseline for comparison). Three independent experiments were performed in eight replicates.

To corroborate the reversibility data, we investigated whether the treatment with oleoresin or hydroalcoholic extract in infected BeWo and HTR8/SVneo cells would interfere with the ability of these parasites to invade and replicate within new fresh cells. Briefly, BeWo and HTR8/SVneo cells were seeded at 1.0x10^6^ cells/well in 6-well plates. After 24 h, cells were infected with *T. gondii* tachyzoites (3:1) for 3 h at 37°C and 5% CO_2_. Then, we treated the cells with oleoresin or hydroalcoholic extract at 16 or 32 µg/mL for BeWo; or 8 or 16 µg/mL for HTR8/SVneo. Either SDZ + PYR or only medium was added as controls. After 24 h, we collected the intracellular parasites from infected cells by multiple passages through a 21- and 26-gauge needle. Finally, *T. gondii* tachyzoites from each experimental condition were then allowed to reinfect previously seeded BeWo (3×10^4^ cells/well/200 µL) and HTR8/SVneo (1.5×10^4^ cells/well/200 µL) cells monolayers in 96-well plates. The ability to infect the cells was analyzed after 3 h, when the β-galactosidase assay was used to quantify the total number of tachyzoites (% of *T. gondii* invasion), as described above. Three independent experiments were performed in eight replicates.

### Adhesion assay of *T. gondii*: pretreated tachyzoites with oleoresin or hydroalcoholic extract

We performed an adherence test (Oliveira et al., 2006; Borges et al., 2016; Teixeira et al., 2020). Cells were seeded at a density of 1.0 × 10^5^ (BeWo) and 6.0 × 10^4^ (HTR8/SVneo) in 24-well plates containing 13-mm coverslips in each well. Cells were fixed with paraformaldehyde (4%) for 30 min. They were then washed three times with 1x phosphate buffered saline (PBS). Subsequently, *T. gondii* tachyzoites were preincubated for 1 h with oleoresin, hydroalcoholic extract or SDZ + PYR. Pretreated tachyzoites with 16 or 32 µg/ml of oleoresin or hydroalcoholic extract were used to adhere BeWo cells; while preincubated parasites with 8 or 16 µg/ml of oleoresin or hydroalcoholic extract were used to adhere HTR8/SVneo cells. SDZ + PYR (200 + 8 μg/mL for later adhesion in BeWo, or 100 + 4 μg/mL for later adhesion in HTR8/SVneo), or only medium, were used as controls. Pretreated tachyzoites by 1 h, as described, were resuspended in medium and incubated with fixed cells for 3 h. Subsequently, the coverslips were incubated overnight with mouse anti-*T. gondii* monoclonal primary antibody [SAG1/p30] (Abcam TP3 #ab8313) (diluted 1:500 in PGN-0.01% saponin solution). Coverslips were then rinsed three times with 1x PBS and incubated with Alexa Fluor 488 conjugated anti-mouse IgG (Invitrogen, USA #A11001) (diluted 1:500 in PGN-0.01% saponin solution), phalloidin conjugated with tetramethylrhodamine isothiocyanate (TRITC) (Sigma, P1951) (diluted 1:50 in PGN+saponin) and TOPRO-3 Iodide (Life Techonologies) (diluted 1:500 in PGN+saponin) for 1 h in the dark at room temperature to label tachyzoites, F-actin and nuclei, respectively. We mounted coverslips on glass slides and samples were analyzed by confocal fluorescence microscopy (Zeiss, LSM 510 Meta, Germany) with an inverted microscope (Zeiss Axiovert 200 M). We analyzed the following parameters: the number of BeWo and HTR8/SVneo cells with adhered tachyzoites and the total number of adhered tachyzoites per cell in a total of 20 randomly chosen fields. Two independent experiments were performed in three replicates.

### Invasion and *T. gondii* intracellular proliferation in cells infected with pretreated tachyzoites with oleoresin or hydroalcoholic extract from *C. multijuga*

After checking the adhesion of pretreated parasites on BeWo and HTR8/SVneo cells, we also investigated the intracellular replication and invasion of pretreated parasites with oleoresins or hydroalcoholic extract (Castanheira et al., 2015; Teixeira et al., 2020). Then, *T. gondii* tachyzoites were preincubated for 1 h at 37°C and 5% CO_2_ with oleoresin or ethanol extract (32 or 16 µg/mL for later infection in BeWo, or 16 or 8 µg/mL for later infection in HTR8/SVneo). In parallel, tachyzoites were also treated with SDZ + PYR (200 + 8 μg/mL for later infection in BeWo, or 100 + 4 μg/mL for later infection in HTR8/SVneo), or only medium. Subsequently, the pretreated parasites were added in previously adhered BeWo or HTR8/SVneo cells in 96-well plates. Finally, two different analyses were performed: (1) to verify the *T. gondii* invasion, both cells were maintained with pretreated tachyzoites for only 3 h; or (2) to verify parasite intracellular proliferation, the cell monolayers were washed after 3 h of infection, a fresh supplemented culture medium was added, and the culture maintained for another 24 h at 37°C and 5% CO_2_. In both experiments, intracellular *T. gondii* was quantified using the β-galactosidase assay, as described above (Silva et al., 2017; Souza et al., 2021). Three independent experiments were performed in eight replicates.

### Viability of human villous explants treated with oleoresin or hydroalcoholic extract

Toxicity of oleoresin and hydroalcoholic extract in human villous explants was performed using viability assays with lactate dehydrogenase (LDH) and MTT, according to previous protocols (Teixeira et al., 2020). In both assays, explants were treated with oleoresin or hydroalcoholic extract from *C. multijuga* at 64, 128 or 256 µg/mL or SDZ + PYR (150 + 200 µg/mL) (Silva et al., 2017). As viability control, explants were treated with culture medium alone. After 24 h of incubation, the culture supernatants were collected and LDH concentration measured according to the manufacturer’s instructions (Castro-Filice et al., 2014), with minor modifications (LDH Liquiform, Labtest Diagnostica S.A., Lagoa Santa, MG, Brazil). This assay is based on the consumption and decreased absorption of NADH at 340 nm, measured by a microplate reader (Versa Max ELISA Microplate Reader, Molecular Devices, Sunnyvale, CA, USA). LDH released in the culture the medium was expressed in U/L of LDH enzymatic activity and was used as a marker of tissue integrity. In parallel, in the same experimental condition, as described above, the viability of the tissue was also evaluated by the MTT test (Teixeira et al., 2020). Tissue viability was expressed in percentages (% viability by MTT incorporation), and the absorbance of villi incubated with culture medium alone (untreated villous explants) was considered to be 100% viable. In addition, we performed a morphological analysis of the treated explants. Tissue sections were included in paraffin, stained with hematoxylin/eosin and examined under a light microscope (BX40 Olympus, Tokyo, Japan). Two independent experiments were performed in six replicates.

### *T. gondii* infection in human villous explants treated with oleoresin or hydroalcoholic extract

Also, we quantified the *T. gondii* intracellular proliferation in explants treated with oleoresin or hydroalcoholic extract from *C. multijuga* using the β-galactosidase colorimetric assay (Silva et al., 2017; Souza et al., 2021), with minor modifications. Explants were cultured in 96-well microplates (one villus per well/200 µl) in supplemented culture medium for 24 h at 37°C and 5% CO_2_. Then, the villi were infected with *T. gondii* tachyzoites (1 × 10^6^ parasites per well/200 µL) and incubated for 24 h at 37°C and 5% CO_2_. Subsequently, the villous explants were thoroughly rinsed with culture medium to remove non-adherent parasites. Following our tissue viability data (LDH and MTT assays), the villi were treated for additional 24 h with oleoresin or hydroalcoholic extract (256 and 128 µg/mL), or SDZ + PYR (150 + 200 µg/mL). Uninfected and untreated or infected and untreated explants were cultured with culture medium alone. Finally, the culture supernatants were collected and stored at -80°C for future cytokine measurement. In addition, the villous explants were collected and stored at −80°C for the following analyses: determination of protein content using Bradford reagent and *T. gondii* intracellular proliferation by β-galactosidase assay. Initially, frozen villous explants were homogenized by the addition of 150 µL of radioimmunoprecipitation assay buffer (RIPA) [50 mM Tris-HCl, 150 mM NaCl, 1% Triton X-100, 1% (w/v) sodium deoxycholate and 0.1% (w/v) sodium deoxycholate SDS, pH 7.5] containing protease inhibitor cocktail (Complete, Roche Diagnostic, Mannheim, Germany). Homogenate was centrifuged at 21,000 × g for 15 min at 4°C, and the supernatant was collected to measure the total amount of protein using the Bradford method (Bradford, 1976). *T. gondii* intracellular proliferation was carried out with 20 µL of supernatants from each sample (Teixeira et al., 2020), absorbance measured at 570 nm using a kinetic plate reader (Versa Max ELISA Microplate Reader, Molecular Devices, Sunnyvale, CA, USA), the number of tachyzoites was normalized according to total protein concentration (µg/mL) of each villus obtained by Bradford assay, and expressed by the number of parasites per µg of tissue. The data were presented as a percentage (% of *T. gondii* proliferation), as described above. Four independent experiments were performed in six replicates.

### Cytokine measurement

We measured cytokines in supernatants using a double-antibody sandwich enzyme-linked immunosorbent assay (ELISA). ELISA for IL-6, IL-8, TNF, IL-10, and MIF were purchased from OpTEIA, BD Bioscience, San Diego, CA, USA; or Duoset R&D Systems, Minneapolis, MN, USA, and tests were performed according to the manufacturer’s instructions. The detection limits of each cytokine were 4.7 pg/mL for IL-6; 31.2 pg/mL for IL-8; 7.8 pg/mL for TNF, IL-10 and MIF.

For villous explants, the data were normalized according to the total protein of each villous. Then, the data about cytokines were obtained by the ratio between concentration of cytokines (pg/mL) and concentration of total protein from Bradford assay (µg/mL), resulting in pg/mg of tissue (Silva et al., 2017; Souza et al., 2021).

### Measurement of intracellular reactive oxygen species

The influence of oleoresin and hydroalcoholic extract in ROS production was also verified in BeWo and HTR8/SVneo cells. The assay was based on the peroxide-dependent oxidation of 2’,7’-dichlorodihydrofluorescein diacetate (H2DCF-DA) to form the fluorescent compound 2’,7’-dichlorofluorescein (DCF), with some modifications. Briefly, BeWo (3×10^4^ cells/well/200 µL) or HTR8/SVneo (1.5×10^4^ cells/well/200 µL) cells were seeded in 96-well black clear-bottom plate. Then, cells were infected or not with *T. gondii* tachyzoites (3:1) for 3 h; they were then washed abundantly with culture medium and treated with oleoresin or hydroalcoholic extract (16 or 32 µg/mL) for BeWo and (8 or 16 µg/mL) for HTR8/SVneo cells, or both cells received SDZ + PYR cells (200 + 8 μg/mL for BeWo, and 100 + 4 μg/mL for HTR8/SVneo) or only medium, as controls. Hydrogen peroxide (H2O2) was used as a positive control for ROS production. After treatment for 24 h, cells were harvested, washed with 1×PBS and incubated with 150 μL of H2DCF-DA (10 μM; diluted in 1×PBS containing 10% FBS) for 45 min at 37°C and 5% CO2 in the dark. Finally, the DCF fluorescence intensity was detected using a microplate reader (GloMax^®^ Discover System, Promega Corporation) at excitation and emission wavelengths of 488 nm and 522 nm, respectively. The data are presented as median fluorescence intensity (MFI). Three independent experiments were performed in eight replicates.

### Ethics

The present research protocol using human tissue samples was approved by the Ethics Committee of the Federal University of Uberlandia, MG, Brazil, with approval number 3.679.426. A consent term was obtained from all subjects and/or their legal guardians.

### Statistical analysis

All data were expressed as means ± standard deviations (SD) using GraphPad Prisma version 8.01. Significant differences were compared to controls by using One-way ANOVA, Bonferroni’s multiple comparisons post-test for the parametric data. Nonparametric data were analyzed by the Kruskal–Wallis test and Tukey’s multiple comparison post-test. Data were considered statistically significant when *P* < 0.05.

## Results

### Leaf hydroalcoholic extract and oleoresin from *C. multijuga* altered the cell viability at higher concentrations

Firstly, we evaluated the cell viability in BeWo and HTR8/SVneo cells treated with hydroalcoholic extract or oleoresin in several concentrations.

**Figure 1:**
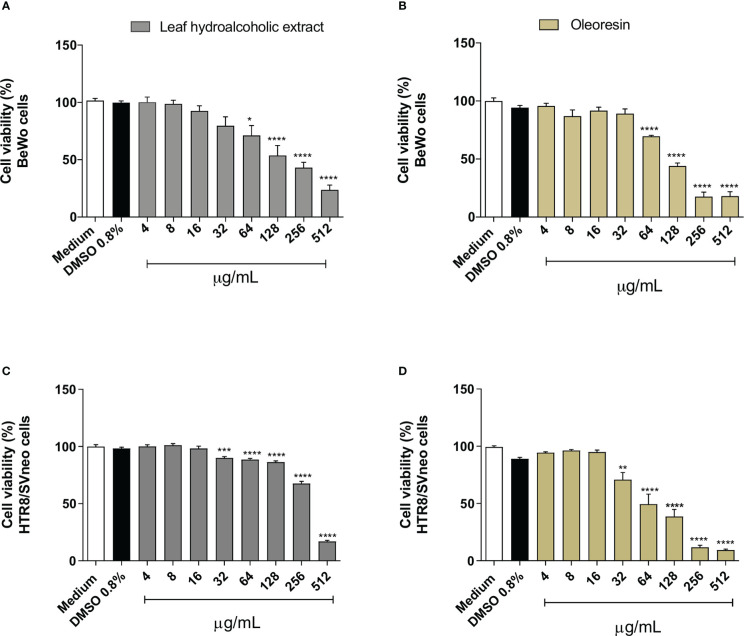
Cell viability. BeWo **(A, B)** and HTR8/SVneo **(C, D)** cells were treated or not with several concentrations of oleoresin or hydroalcoholic extract from *C. multijuga* for 24 h, and MTT assay was performed. Cell viability was expressed as percentage (cell viability %), considering the absorbance of cells incubated only with the medium as 100% viability. The results are expressed as means ± standard deviation of three independent experiments performed with eight replicates. Significant differences detected by One-Way ANOVA with Bonferroni’s multiple comparison post-test, or Kruskal-Wallis when appropriate. ^*^ *P* < 0.05, ^**^ *P* < 0.001, ^***^ *P* < 0.0001, and ^****^ *P* < 0.00001 in relation to the medium.

Reduced cell viability was detected in BeWo cells exposed to hydroalcoholic extract or oleoresin from 64 to 512 µg/mL (^*^ *P* < 0.05, ^****^ *P* < 0.00001) in comparison to untreated cells (medium) (**Figures 1A, B**). DMSO-treated cells did not show any change in cell viability in relation to untreated cells (**Figures 1A, B**). On the other hand, HTR8/SVneo diminished cell viability when treated with hydroalcoholic extract or oleoresin from 32 (^***^ *P* < 0.0001, ^**^ *P* < 0.001) to 512 µg/mL (^****^ *P* < 0.00001) when compared to untreated cells (medium) (**Figures 1C, D**). As observed for BeWo, DMSO also did not induce change in cell viability for HTR8/SVneo (**Figures 1C, D**).

### Leaf hydroalcoholic extract and oleoresin from *C. multijuga* significantly reduced *T. gondii* intracellular proliferation in BeWo and HTR8/SVneo cells

In our cell viability assay, we established concentrations of hydroalcoholic extract and oleoresin that did not alter the viability of BeWo and HTR8/SVneo cells for the subsequent set up of experiments.

**Figure 2:**
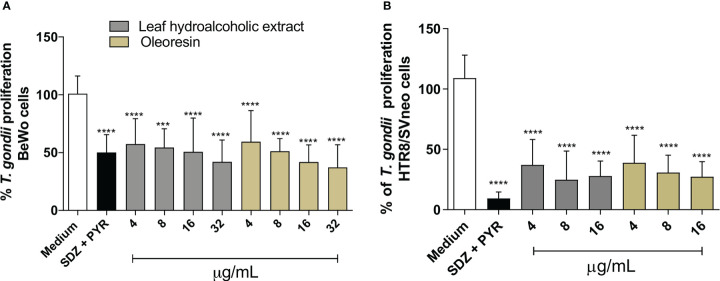
*T. gondii* intracellular proliferation. BeWo **(A)** and HTR8/SVneo **(B)** cells were infected by *T. gondii* and treated or not with non-toxic concentrations of hydroalcoholic extract or oleoresin from *C. multijuga* for 24 h. Untreated and infected cells (medium) were considered as 100% parasite proliferation, and SDZ + PYR was used as positive control. *T. gondii* intracellular proliferation was analyzed using a β-galactosidase colorimetric assay and expressed as percentage (% of *T. gondii* proliferation) in comparison to untreated cells (medium). The results were expressed as means ± standard deviation of three experiments performed in eight replicates. Significant differences detected by One-Way ANOVA, Bonferroni’s multiple comparisons post-test. ^***^ *P* < 0.0001 or ^****^ *P* < 0.00001 in relation to the medium.

Both hydroalcoholic extract and oleoresin reduced significantly the parasite proliferation in BeWo (**Figure 2A**) and HTR8/SVneo (**Figure 2B**) cells in all concentrations tested in comparison to untreated cells (medium) (^***^ *P* < 0.0001; ^*****^ *P* < 0.00001). In addition, sulphadiazine and pyrimethamine (SDZ + PYR) also diminished *T. gondii* replication in relation to untreated cells (^****^ *P* < 0.00001). These results demonstrated that both the hydroalcoholic extract and oleoresin had an antiproliferative effect against *T. gondii*.

### Leaf hydroalcoholic extract and oleoresin exhibited an irreversible antiparasitic effect

In order to determine whether the antiparasitic effects promoted by the hydroalcoholic extract and oleoresin from *C. multijuga* would be irreversible, we exposed infected BeWo and HTR8/SVneo cells to the hydroalcoholic extract or oleoresin for 24 h, then cell monolayers were rinsed and incubated with medium free of treatment for additional 24 h. At the same time, we quantified the parasite proliferation after 24 h of treatment as a baseline for comparison. For BeWo cells, we choose 16 and 32 µg/mL for both the hydroalcoholic extract and oleoresin; for HTR8/SVneo, 8 and 16 µg/mL were the choice.

**Figure 3:**
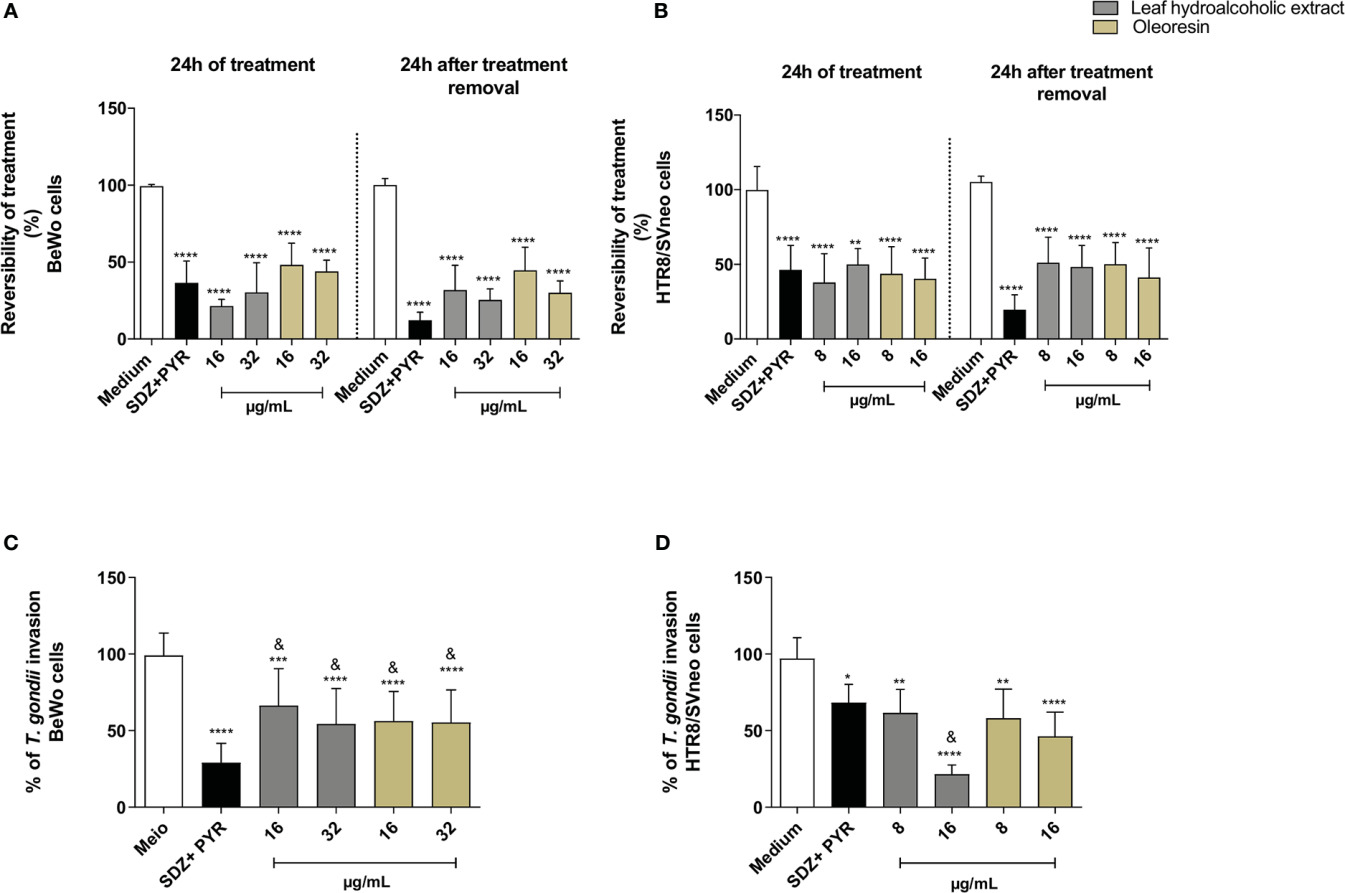
Reversibility test. BeWo **(A)** and HTR8/SVneo **(B)** cells were infected by *T. gondii* and treated or not with non-toxic concentrations of the hydroalcoholic extract or oleoresin from *C. multijuga* for 24 h. In parallel, both cells were infected, treated, washed to remove the treatments, and maintained for additional 24 h in the medium free of treatment. Untreated and infected cells (medium) were considered as 100% reversibility of treatment, and SDZ + PYR was used as positive control. *T. gondii* intracellular proliferation was analyzed using a β-galactosidase colorimetric assay and expressed as percentage (reversibility of treatment) in comparison to untreated cells (medium). The reversibility index measured the ability of parasites to recover from treatment and proliferate in new cells. Finally, parasites obtained directly from BeWo **(C)** and HTR8/SVneo **(D)** cells treated with the hydroalcoholic extract or oleoresin were collected and used to infect new cells for 3 h. The number of tachyzoites was determined using the β-galactosidase assay and expressed as % of *T. gondii* invasion. The results were expressed as means ± standard deviation of three experiments performed in eight replicates. Significant differences detected by the One-Way ANOVA, Bonferroni’s multiple comparisons post-test. ^*^ *P* < 0.05, ^**^ *P* < 0.001, ^***^ *P* < 0.0001 or ^****^ *P* < 0.00001 in relation to the medium. ^&^ *P* < 0.05 in relation to SDZ + PYR.

As previously detected (**Figure 2**), again both the hydroalcoholic extract and oleoresin reduced *T. gondii* intracellular proliferation after 24 h of treatment in BeWo (**Figure 3A**) and HTR8/SVneo cells (**Figure 3B**) when compared to untreated cells (medium) (^**^ *P* < 0.001, ^****^ *P* < 0.00001). Also, SDZ + PYR decreased parasite replication in relation to untreated cells (^****^ *P* < 0.00001) (**Figures 3A, B**). Interestingly, both the hydroalcoholic extract and oleoresin showed an irreversible effect on tachyzoites, since a reduced parasite growth remained active in BeWo and HTR8/SVneo cells in comparison to untreated cells (considered as 100% reversibility), even after removal of treatments for additional 24 h (^****^ *P* < 0.00001) (**Figures 3A, B**). For both cells, SDZ + PYR also showed irreversible effect (^****^ *P* < 0.00001) (**Figures 3A, B**). These results suggest that both the hydroalcoholic extract and oleoresin maintained their antiproliferative effect even after the elimination of the treatment.

In order to obtain more information about the effect triggered by both the hydroalcoholic extract and oleoresin on *T. gondii* tachyzoites, we evaluated whether intracellular parasites obtained directly from treated-BeWo and HTR8/SVneo cells could maintain their ability to infect new host cells. For this purpose, tachyzoites were collected from treated-BeWo and HTR8/SVneo cells and used to infect new monolayer cells. For BeWo cells, all concentrations of hydroalcoholic extract and oleoresin reduced the ability of tachyzoites to infect new BeWo cells when compared to untreated cells (medium) (^***^ *P* < 0.0001; ^****^ *P* < 0.00001) (**Figure 3C**). SDZ + PYR also reduced the ability of parasites to infect new BeWo cells in relation to medium (^****^ *P* < 0.00001) (**Figure 3C**). Although the hydroalcoholic extract and oleoresin have downmodulated the ability of parasites to infect new BeWo cells, this effect was lower if compared to classical treatment (SDZ + PYR) (^&^ *P* < 0.05) (**Figure 3C**). Finally, for HTR8/SVneo cells, all treatments (hydroalcoholic extract, oleoresin and SDZ + PYR) dampened the capacity of *T. gondii* to infect new HTR8/SVneo cells in comparison to untreated cells (^*^ *P* < 0.05; ^**^ *P* < 0.001; ^****^ *P* < 0.00001) (**Figure 3D**). Interestingly, parasites derived from hydroalcoholic extract-treated-cells at 16 µg/mL demonstrated lower ability to infect new cells when compared to SDZ + PYR (^&^ *P* < 0.05) (**Figure 3D**).

These findings indicate that the inhibitory effects promoted by the hydroalcoholic extract and oleoresin from *C. multijuga* dampen the ability of parasites to invade new host cells.

### Pretreatment of *T. gondii* tachyzoites with leaf hydroalcoholic extract and oleoresin altered adhesion, invasion and subsequent intracellular proliferation

To assess whether hydroalcoholic extract and oleoresin have a direct action on tachyzoites, we performed a variety of assays. In the first set of experiments, we evaluated whether the hydroalcoholic extract and oleoresin treatments would affect parasite adhesion to host cells. For this purpose, *T. gondii* tachyzoites were pretreated for 1 h with oleoresin or hydroalcoholic extract and then incubated with previously fixed-BeWo or HTR8/SVneo cells.

**Figure 4:**
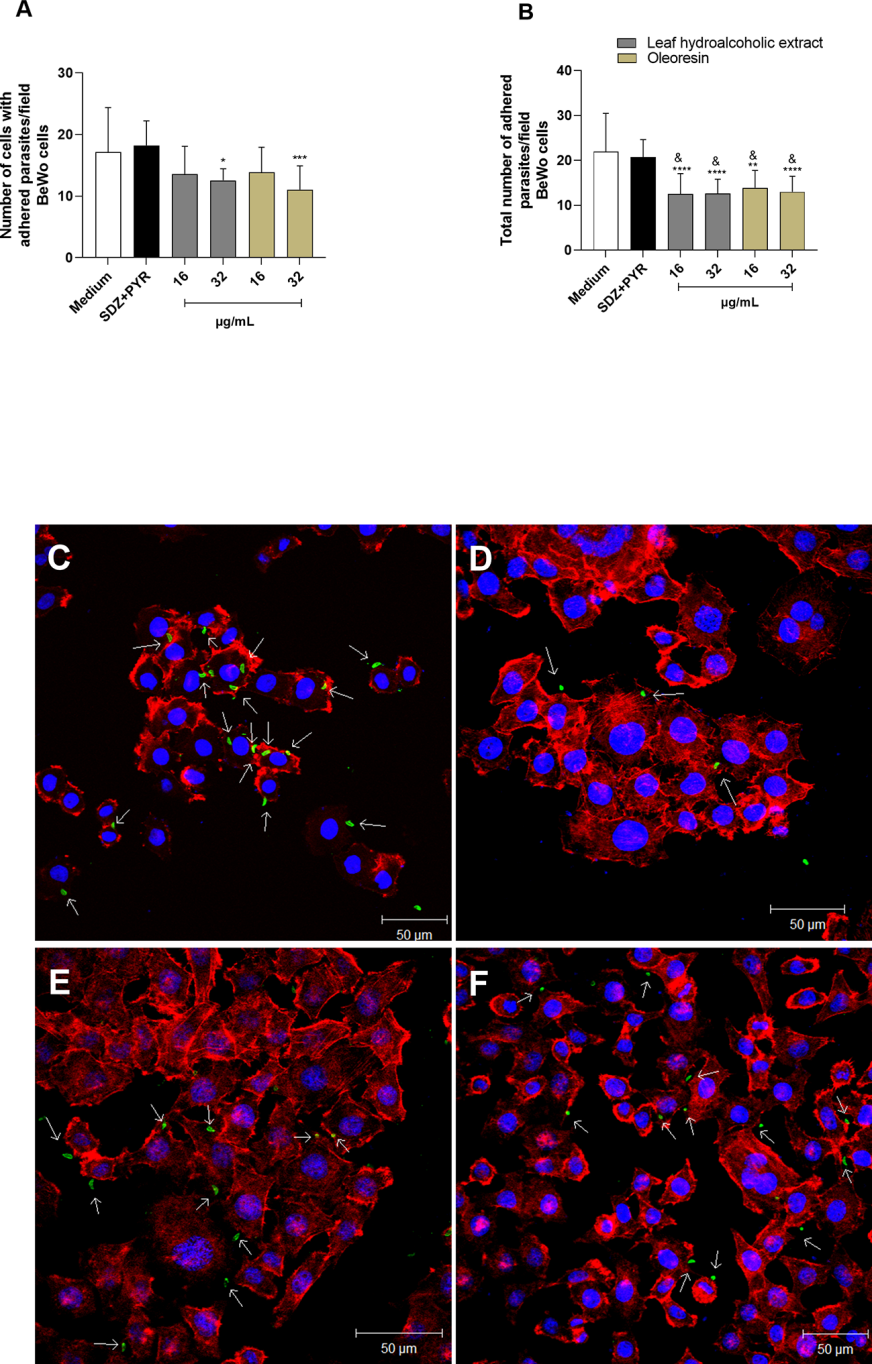
Adhesion assay in BeWo cells. *T. gondii* tachyzoites were preincubated for 1 h with the hydroalcoholic extract or oleoresin from *C. multijuga*, and then allowed to interact with previously fixed BeWo cells during 3 h. As control, tachyzoites were incubated with SDZ + PYR or only medium (medium). The number of cells with adhered parasites **(A)** and the total number of adhered parasites **(B)** were determined in a total of 20 fields examined randomly with 20X objective lens. Representative images highlighting the effect of treatments on the tachyzoite-host cell interaction: **(C)** untreated parasites, **(D)** 32 µg/mL hydroalcoholic extract, **(E)** 16 µg/mL oleoresin and **(F)** 32 µg/mL oleoresin. The results were expressed as means ± standard deviation of two experiment performed in three replicates. Significant differences detected by One-Way ANOVA and Bonferroni’s multiple comparisons post-test. ^*^ *P* < 0.05, **P < 0.001, ^***^ *P* < 0.0001 or ^****^ *P* < 0.00001 in relation to the medium. ^&^ *P* < 0.05 in relation to SDZ + PYR. White arrows indicate tachyzoites attached to BeWo cells. The cell nucleus is labeled with TOPRO-3 (blue). *T. gondii* tachyzoites labeled with Alexa Fluor 488-conjugated anti-mouse IgG (green). Phalloidin-TRITC labeled F-actin is shown in red. Scale bar: 50 µm.

As result for BeWo cells, only the hydroalcoholic extract or oleoresin at 32 µg/mL was able to reduce the number of cells with adhered parasites when compared to untreated parasites (medium) (^***^ *P* < 0.0001, ^*^ *P* < 0.05) (**Figure 4A**). On the other hand, all concentrations of treatments, except for SDZ + PYR, reduced the total number of adhered parasites in relation to untreated parasites (^****^ *P* < 0.00001, ^**^ *P* < 0.001) and SDZ + PYR-treated parasites (^&^ *P* < 0.05) (**Figure 4B**). Representative images showing the effect of the hydroalcoholic extract and oleoresin on BeWo cells incubated with pretreated-tachyzoites can be observed in **Figures 4C-F**.

**Figure 5:**
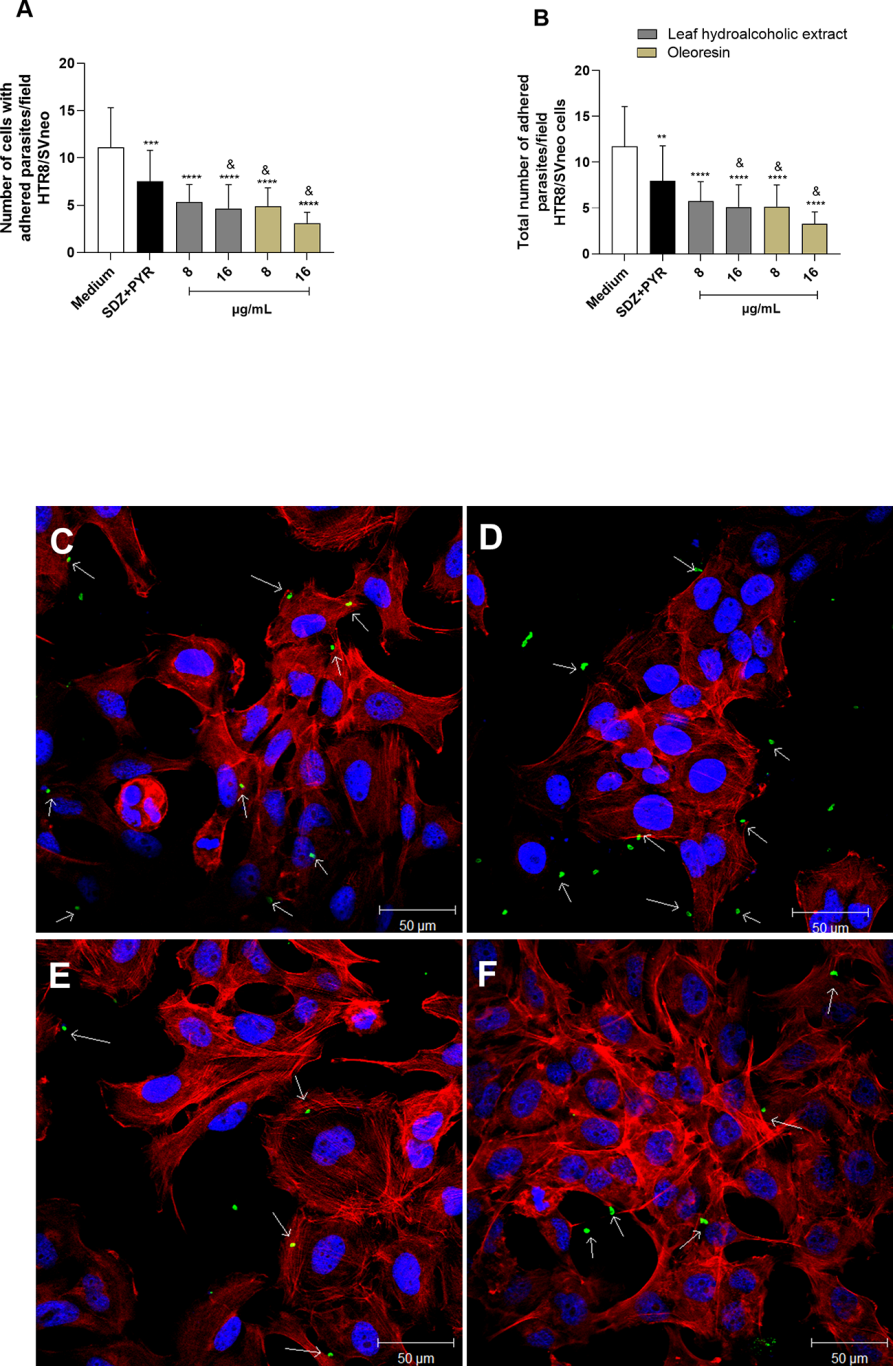
Adhesion assay in HTR8/SVneo cells. *T. gondii* tachyzoites were preincubated for 1 h with the hydroalcoholic extract or oleoresin from *C*. *multijuga*, and then allowed to interact with previously fixed HTR8/SVneo cells during 3 h. As control, tachyzoites were incubated with SDZ + PYR or only medium (medium). The number of cells with adhered parasites **(A)** and the total number of adhered parasites **(B)** were determined in a total of 20 fields examined randomly with 20X objective lens. Representative images highlighting the effect of the treatments on the tachyzoite-host cell interaction: **(C)** untreated parasites, **(D)** 16 µg/mL hydroalcoholic extract, **(E)** 8 µg/mL oleoresin, and **(F)** 16 µg/mL oleoresin. The results were expressed as means ± standard deviation of two experiment performed in three replicates. Significant differences detected by One-Way ANOVA and Bonferroni’s multiple comparisons post-test. ^**^ *P* < 0.001, ^***^ *P* < 0.0001 or ^****^ *P* < 0.00001 in relation to the medium. ^&^ *P* < 0.05 in relation to SDZ + PYR. White arrows indicate tachyzoites attached to HTR8/SVneo cells. The cell nucleus is labeled with TOPRO-3 (blue). *T. gondii* tachyzoites labeled with Alexa Fluor 488-conjugated anti-mouse IgG (green). Phalloidin-TRITC labeled F-actin is shown in red. Scale bar: 50 µm.

Similarly, parasites pretreated with the hydroalcoholic extract or oleoresin diminished their ability to adhere in HTR8/SVneo cells, since the number of cells with adhered parasites as well as the total number of adhered parasites per field were significantly smaller when related to untreated parasites (medium) (^****^ *P* < 0.00001) and SDZ + PYR-treated parasites (^&^ *P* < 0.05) (**Figure 5A, B**). Also, SDZ + PYR reduced the number of cells with adhered parasites (^***^ *P* < 0.0001) and the total number of adhered parasites (^**^ *P* < 0.001) in relation to the medium (**Figure 5A, B**). Representative images showing the effect of the hydroalcoholic extract and oleoresin on HTR8/SVneo cells incubated with pretreated-tachyzoites can be observed in **Figures 5C-F**.

**Figure 6:**
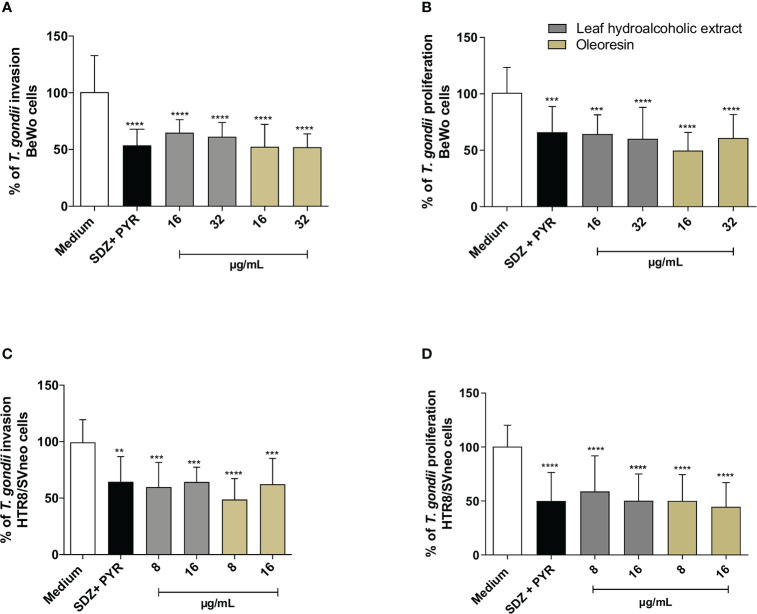
Invasion and proliferation assay. *T. gondii* tachyzoites were preincubated for 1 h with the hydroalcoholic extract or oleoresin from *C. multijuga*, and then allowed to interact with BeWo **(A, B)** or HTR8/SVneo **(C, D)** cells during 3 or 24 h to verify invasion and intracellular proliferation, respectively. As control, tachyzoites were incubated with SDZ + PYR or only medium (medium). The % of *T. gondii* invasion and the % of *T. gondii* proliferation were determined using the β-galactosidase activity. Untreated parasites (medium) were considered as 100% invasion and proliferation. The results were expressed as means ± standard deviation of three experiments performed in eight replicates. Significant differences detected by One-Way ANOVA and Bonferroni’s multiple comparisons post-test. ^**^ *P* < 0.001, ^***^ *P* < 0.0001 or ^****^ *P* < 0.00001 in relation to the medium.

Considering that pretreated parasites had lower ability to adhere to host cells (**Figures 4**, **5**), we now verified the capacity to invade and proliferate when also preincubated with the hydroalcoholic extract or oleoresin. Our data demonstrated that the pretreatment of *T. gondii* tachyzoites with all the hydroalcoholic extract or oleoresin concentrations triggered low rates of invasion and intracellular proliferation, regardless of the cell type (^***^ *P* < 0.0001, ^****^ *P* < 0.00001), in comparison to untreated parasites (medium) (**Figures 6A-D**). SDZ + PYR also reduced the invasion and replication of pretreated tachyzoites in BeWo and HTR8/SVneo cells in relation to the medium (^**^ *P* < 0.001, ^***^ *P* < 0.0001, ^****^ *P* < 0.00001) (**Figures 6A-D**).

These findings indicate that the hydroalcoholic extract and oleoresin from *C. multijuga* dampen the ability of parasites to adhere, invade and proliferate in host cells from maternal-fetal interface.

### Leaf hydroalcoholic extract and oleoresin from *C. multijuga* upregulated IL-6 and downmodulated IL-8 in BeWo cells

So far, our findings demonstrated that the hydroalcoholic extract and oleoresin from *C. multijuga* have a direct effect on the parasites, since treated parasites have reduced ability to adhere, invade and replicate into the cells. However, we could not exclude the possibility that these compounds may affect the host cell environment. Thus, we investigated the possible immunomodulatory effects of the hydroalcoholic extract and oleoresin by measuring cytokines in culture supernatant.

**Figure 7:**
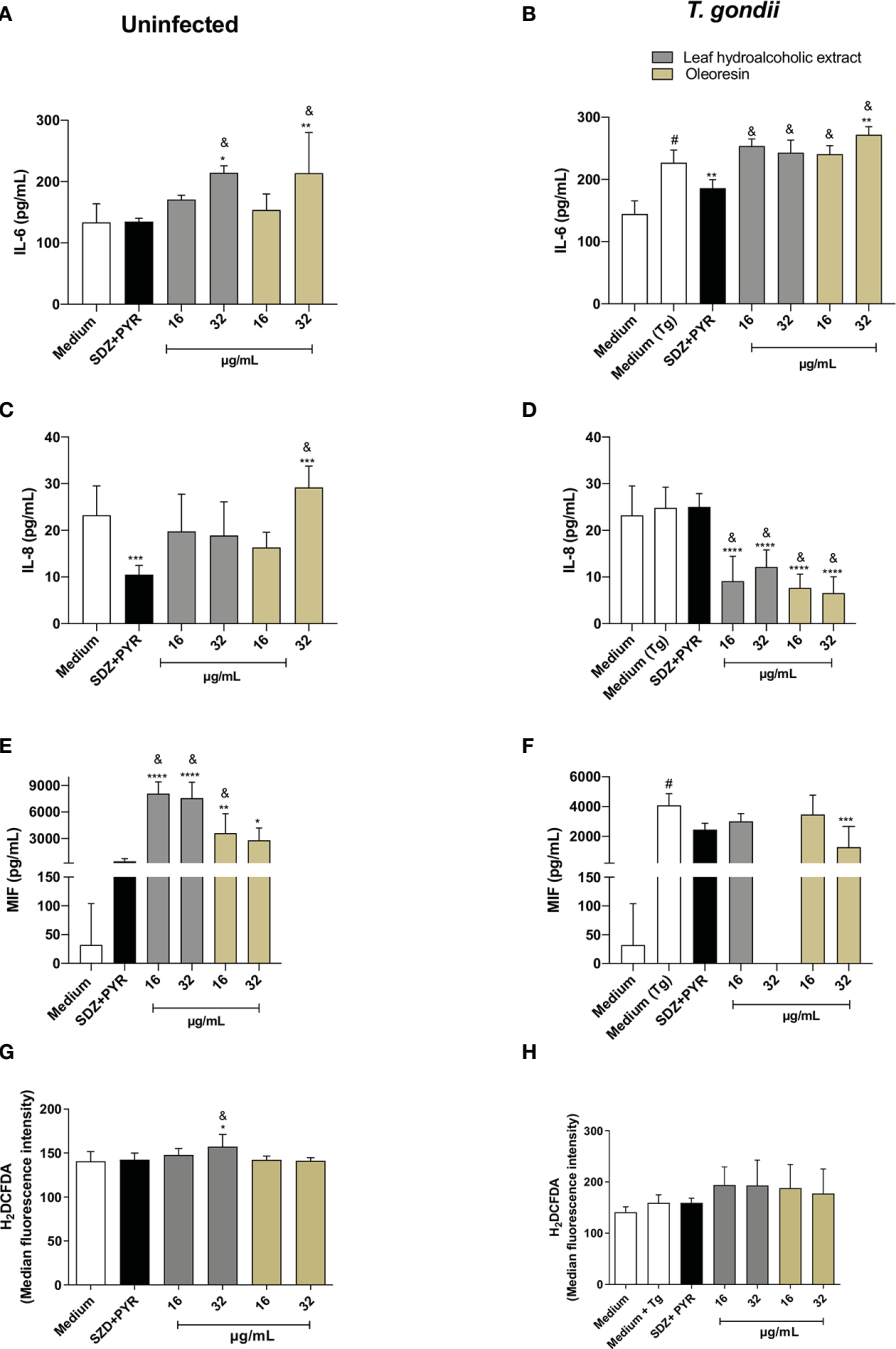
Cytokine and ROS production in BeWo cells. BeWo cells were infected and treated or not with the hydroalcoholic extract or oleoresin from *C. multijuga* for 24 h. Untreated and uninfected cells (medium), untreated and infected cells (medium Tg) and SDZ + PYR were used as controls. Then, supernatants were collected and used to measure IL-6 **(A, B)**, IL-8 **(C, D)** and MIF **(E, F)**. In parallel, BeWo cells were infected, treated or not with the hydroalcoholic extract or oleoresin from *C. multijuga* for 24 h, incubated with the probe 2′,7′-dichlorodihydrofluorescein diacetate (H2DCF-DA), ROS production was measured by a plate reader and data expressed as mean fluorescence intensity (MFI) **(G, H)**. The results were expressed as means ± standard deviation of three experiments performed in eight replicates. Significant differences detected by One-Way ANOVA, Bonferroni’s multiple comparisons post-test. ^*^ *P* < 0.01, ^**^ *P* < 0.001, ^***^ *P* < 0.0001 or ^****^ *P* < 0.00001 in relation to medium (for uninfected cells) or to medium Tg (for infected cells). ^&^ *P* < 0.05 in relation to SDZ + PYR (for uninfected or infected cells). ^#^ *P* < 0.05 between medium and medium Tg.

For BeWo cells, our results showed that in the absence of infection, concentrations of 32 µg/mL of the hydroalcoholic extract and oleoresin induced an increase in IL-6 levels in comparison to untreated cells (medium) (^*^ *P* < 0.05, ^**^ *P* < 0.001) and SDZ + PYR (^&^ *P* < 0.05) (**Figure 7A**). After *T. gondii* infection, levels of IL-6 were higher in untreated/infected cells (medium Tg) compared to untreated and uninfected cells (medium) (^#^ *P* < 0.05). However, SDZ + PYR reduced IL-6 in relation to untreated/infected cells (^**^ *P* < 0.001). In addition, all concentrations of the hydroalcoholic extract or oleoresin induced an upregulation of IL-6 in infected BeWo cells when compared to SDZ + PYR-treated cells (^&^ *P* < 0.05). Also, 32 µg/mL of oleoresin induced an increase of IL-6 in comparison to untreated/infected cells (^**^ *P* < 0.001) (**Figure 7B**).

Regarding IL-8, uninfected BeWo cells reduced or increased the level of this cytokine when treated with SDZ + PYR or 32 µg/mL, respectively (^***^ *P* < 0.0001) in relation to untreated cells (medium) (**Figure 7C**). Interestingly, 32 µg/mL oleoresin triggered a higher release of IL-8 in comparison to SDZ + PYR-treated cells (^&^ *P* < 0.05) (**Figure 7C**). However, all concentrations of the hydroalcoholic extract and oleoresin downmodulated IL-8 in infected BeWo cells in comparison to untreated/infected cells (medium Tg) (^****^ *P* < 0.00001) or SDZ + PYR-treated cells (^&^ *P* < 0.05) (**Figure 7D**).

For MIF production, uninfected BeWo cells augmented the release of this cytokine in all concentrations of the hydroalcoholic extract and oleoresin when compared to untreated cells (medium) (^*^ *P* < 0.01, ^**^ *P* < 0.001, ^****^ *P* < 0.00001) or SDZ + PYR-treated cells (^&^ *P* < 0.05) (**Figure 7E**). However, in the presence of infection, untreated and 32 µg/mL oleoresin-treated cells upregulated or downmodulated MIF in comparison to medium (^#^ *P* < 0.05) or medium Tg (^***^ *P* < 0.0001), respectively (**Figure 7F**).

Finally, we measured ROS in BeWo cells infected or not with *T. gondii* and treated or not with the hydroalcoholic extract or oleoresin. We just observed that uninfected cells treated with 32 µg/mL of the hydroalcoholic extract augmented the level of ROS in relation to untreated cells (medium) (^*^ *P* < 0.01) and SDZ + PYR-treated cells (^&^ *P* < 0.05) (**Figure 7G**). Additionally, infected BeWo cells did not change ROS release in presence of any treatment (**Figure 7H**).

TNF and IL-10 were not detected in BeWo cell supernatants under any experimental conditions (data not shown).

### Leaf hydroalcoholic extract and oleoresin from *C. multijuga* slightly modulated cytokine and ROS production in HTR8/SVneo cells

We also verified cytokine and ROS production in HTR8/SVneo cells infected or not with *T. gondii* and treated with the hydroalcoholic extract or oleoresin.

**Figure 8:**
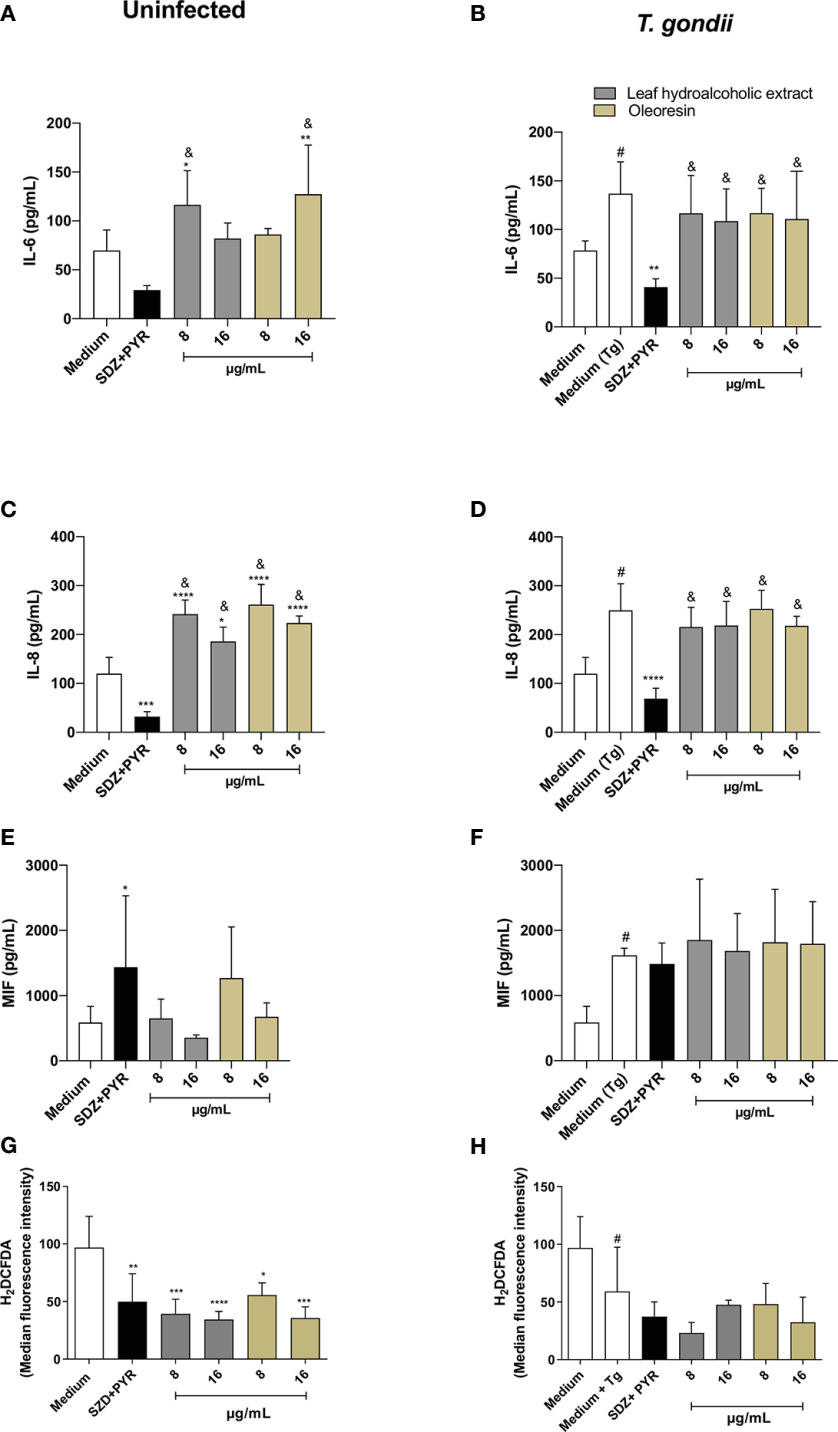
Cytokine and ROS production in HTR8/SVneo cells. HTR8/SVneo cells were infected and treated or not with the hydroalcoholic extract or oleoresin from *C. multijuga* for 24 h. Untreated and uninfected cells (medium), untreated and infected cells (medium Tg) and SDZ + PYR were used as controls. Then, supernatants were collected and used to measure IL-6 **(A, B)**, IL-8 **(C, D)** and MIF **(E, F)**. In parallel, HTR8/SVneo cells were infected, treated or not with the hydroalcoholic extract or oleoresin from *C. multijuga* for 24 h, incubated with the probe 2′,7′-dichlorodihydrofluorescein diacetate (H2DCF-DA), ROS production was measured by a plate reader and data were expressed as mean fluorescence intensity (MFI) **(G, H)**. The results were expressed as means ± standard deviation of three experiments performed in eight replicates. Significant differences detected by One-Way ANOVA, Bonferroni’s multiple comparisons post-test. ^*^ *P* < 0.01, ^**^ *P* < 0.001, ^***^ *P* < 0.0001 or ^****^ *P* < 0.00001 in relation to the medium (for uninfected cells) or to the medium Tg (for infected cells). ^&^ *P* < 0.05 in relation to SDZ + PYR (for uninfected or infected cells). ^#^ *P* < 0.05 between medium and medium Tg.

In the absence of infection, HTR8/SVneo cells increased IL-6 release when treated with the hydroalcoholic extract (8 µg/mL) or oleoresin (16 µg/mL) in comparison to untreated cells (medium) (^*^ *P* < 0.01, ^**^ *P* < 0.001) and SDZ + PYR-treated cells (^&^ *P* < 0.05) (**Figure 8A**). In the presence of infection, untreated HTR8/SVneo cells, as well as cells treated with the hydroalcoholic extract or oleoresin, increased the levels of IL-6 in relation to the medium or SDZ + PYR-treated cells, respectively (^#^ *P* < 0.05, ^&^ *P* < 0.05) (**Figure 8B**). Also, IL-6 was lower in SDZ + PYR-treated cells in comparison to untreated and infected cells (medium Tg) (^**^ *P* < 0.001) (**Figure 8B**).

Regarding IL-8 for uninfected cells, SDZ + PYR reduced IL-8 (^***^ *P* < 0.0001) and all concentrations of the hydroalcoholic extract and oleoresin (^*^ *P* < 0.01, ^****^ *P* < 0.00001) increased IL-8 when compared to the medium (**Figure 8C**). Also, the hydroalcoholic extract and oleoresin presented higher levels of IL-8 in comparison to SDZ + PYR (^&^ *P* < 0.05) (**Figure 8C**). When HTR8/SVneo cells were infected by *T. gondii*, IL-8 was augmented in relation to the medium (^#^ *P* < 0.05), SDZ + PYR decreased IL-8 in comparison to only infected cells (medium Tg) (^****^ *P* < 0.00001), and all treatments with the hydroalcoholic extract or oleoresin upregulated IL-8 in relation to SDZ + PYR (^&^ *P* < 0.05) (**Figure 8D**).

The MIF production was not significantly changed in uninfected HTR8/SVneo cells, except for uninfected and SDZ + PYR-treated cells (^*^ *P* < 0.01) (**Figure 8E**). Although not statistically different, the hydroalcoholic extract and oleoresin showed an increase in MIF release in infected HTR8/SVneo cells, while only infected cells augmented MIF in comparison to untreated and uninfected cells (^#^ *P* < 0.05) (**Figure 8F**).

Finally, we measured ROS in HTR8/SVneo cells infected or not with *T. gondii* and treated or not with the hydroalcoholic extract or oleoresin. We observed that uninfected cells treated with the hydroalcoholic extract, oleoresin or SDZ + PYR presented low levels of ROS in relation to untreated cells (medium) (^*^ *P* < 0.01, ^**^ *P* < 0.001, ^***^ *P* < 0.0001, ^****^ *P* < 0.00001) (**Figure 8G**). Although not statistically different, the hydroalcoholic extract and oleoresin showed a reduction in ROS release in infected HTR8/SVneo cells, while only infected cells diminished ROS in comparison to untreated and uninfected cells (^#^ *P* < 0.05) (**Figure 8H**).

TNF and IL-10 were not detected in supernatants under any experimental conditions (data not shown).

### Human chorionic villous explants treated with the hydroalcoholic extract or oleoresin did not alter viability

**Figure 9:**
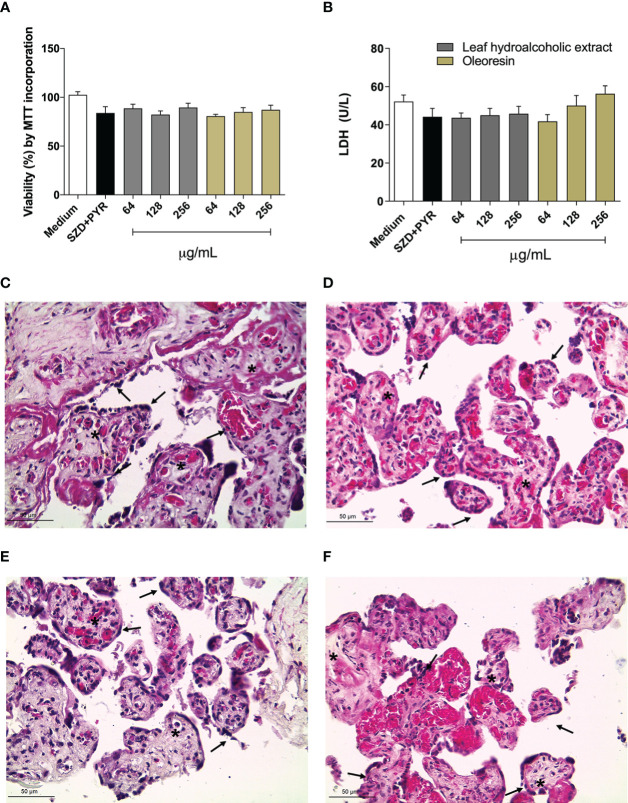
Viability of human villous explants. The villous explants were treated for 24 h with hydroalcoholic extract or oleoresin from *C*. *multijuga*, SDZ+PYR or culture medium alone (medium). **(A)** Tissue viability is shown in percentages (% viability by incorporation of MTT). **(B)** Supernatants were collected and used to measure LDH levels (U/L). Representative images of villi incubated with **(C)** culture medium alone, **(D)** SDZ+PYR, 256 µg/mL hydroalcoholic extract **(E)**, 256 µg/mL oleoresin **(F)**. Data are expressed as means ± standard deviation of two experiments performed in six replicates. Significant differences detected by One-way ANOVA, Bonferroni’s multiple comparisons post-test (statistically significant when *P* < 0.05). Hematoxylin-eosin (HE) stained histological sections show syncytiotrophoblast cells (black arrows) and mesenchyme (*). Scale bar: 50 µm.

First, to determine non-toxic concentration to use in the experiments, the villous explant viability after treatments with oleoresin or hydroalcoholic extract was performed by measuring LDH release and MTT assay. Our data showed that both oleoresin and hydroalcoholic extract did not alter tissue viability at any of the concentrations tested when compared to untreated explants (medium). Also, treatment with SDZ + PYR did not cause significant cytotoxicity in villous explants in relation to the untreated group (**Figures 9A, B**). In addition, treatments did not alter the tissue morphological structure, which was highlighted by the typical morphology of syncytiotrophoblast cells (black arrows) and mesenchyme (*) compared to untreated villous explants (**Figures 9C-F**).

### The hydroalcoholic extract and oleoresin from *C. multijuga* reduced parasite intracellular proliferation, but did not alter the cytokine profile in human chorionic villous explants

**Figure 10:**
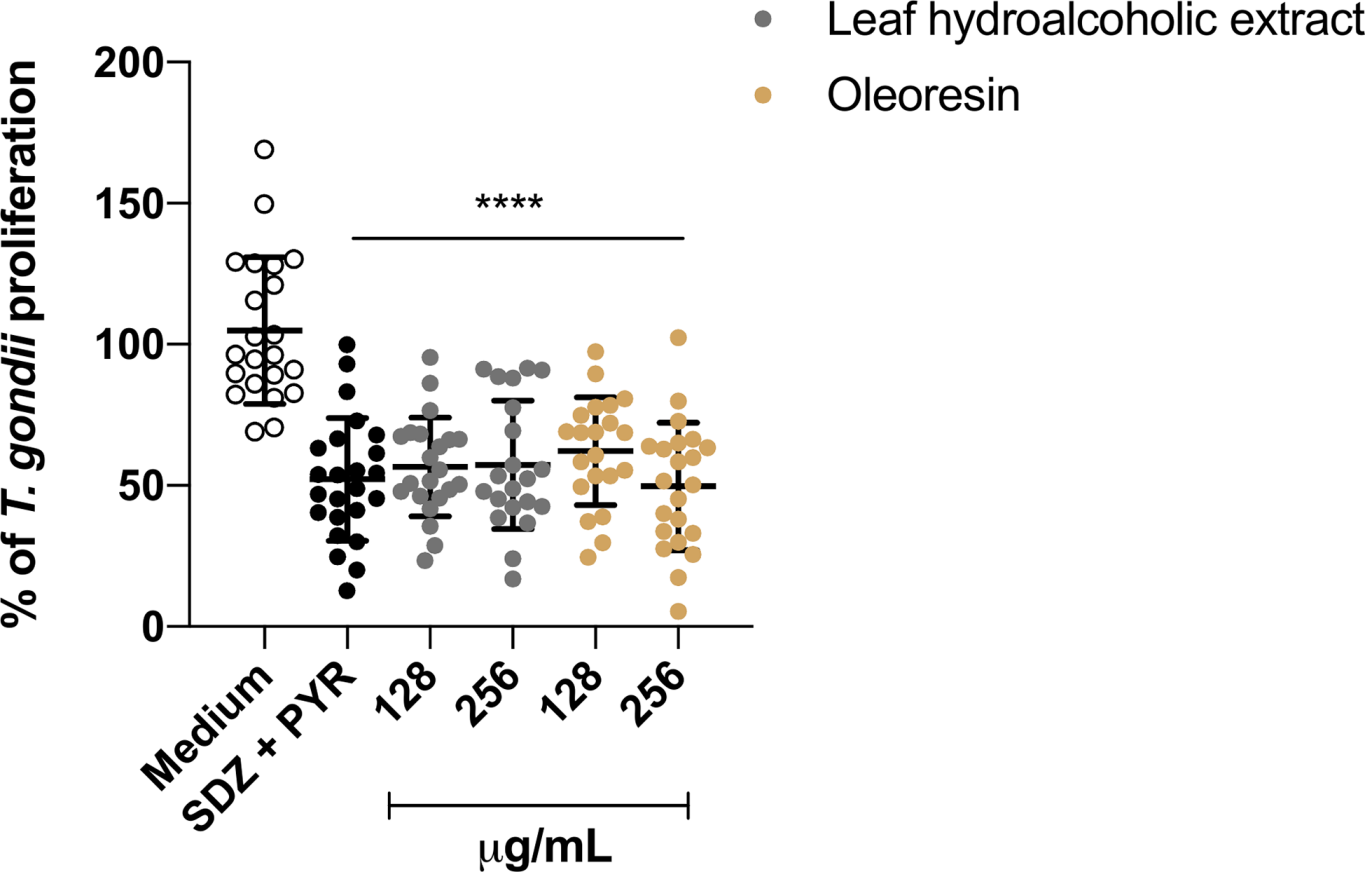
*T. gondii* intracellular proliferation. The human villous explants were infected with *T. gondii* followed by treatment for an additional 24 h with hydroalcoholic extract, oleoresin, SDZ+PYR or culture medium alone (medium). *T. gondii* intracellular proliferation was measured by β-galactosidase assay, and the number of tachyzoites was expressed as a percentage (% of *T. gondii* proliferation), and the untreated/infected (medium) was considered as 100% parasite proliferation. Data are expressed as means ± standard deviation of four experiments performed in six replicates. ^*^Comparison with infected/untreated cells (^****^ *P* < 0.00001). Significant differences detected by One-way ANOVA, Bonferroni’s multiple comparisons post-test differences were considered when *P* < 0.05.

We evaluated the effects of the hydroalcoholic extract and oleoresin (256 and 128 µg/mL) on the control of *T. gondii* intracellular proliferation in human chorionic villi using the β-galactosidase assay. We observed that treatments at both concentrations significantly reduced the percentage of intracellular parasites in comparison to untreated/infected explants (considered as 100% proliferation) (^****^ *P* < 0.00001). As expected, treatment with SDZ + PYR also inhibited parasite growth (^****^ *P* < 0.00001) when compared to the untreated group (medium) (**Figure 10**).

In addition, we evaluated whether the hydroalcoholic extract and oleoresin would modulate the cytokine profile in chorionic villi. Our results showed that IL-6, IL-8, MIF and TNF showed no significant change in their production when uninfected explants were treated with both hydroalcoholic extract or oleoresin if compared to medium, except for TNF when 256 µg/mL hydroalcoholic extract was added in the samples (^*^ *P* < 0.05) (**Supplementary Figure 1**). Finally, *T. gondii* infection reduced the levels of all cytokines in comparison to untreated explants (medium), regardless of hydroalcoholic extract and oleoresin treatments (^#^ *P* < 0.05) (**Supplementary Figure 1**).

## Discussion

Toxoplasmosis is considered as a global health problem with nearly a billion people infected, reaching a prevalence of the disease in 71.5% (Ahmadpour et al., 2019). Regarding the treatment, a combination of sulfadiazine and pyrimethamine (SDZ + PYR) is the first choice to treat congenital toxoplasmosis, one of the most serious forms of the disease (Abugri et al., 2019). Despite the great clinical importance, the conventional treatment for congenital toxoplasmosis is currently limited, and it is also linked to serious side effects in both the mother and the child (Montoya and Remington, 2008; Halonen and Weiss, 2013). In this sense, the search for alternative therapeutic tools has gained attention.

In order to assess the impact of both the leaf hydroalcoholic extract and oleoresin from *C. multijuga* in the *T. gondii* infection, we used BeWo and HTR8/SVneo cells as villous and extravillous trophoblast cell models, respectively, being considered as excellent experimental models for the study of congenital toxoplasmosis *in vitro* (Barbosa et al., 2014; Souza et al., 2021; Teixeira et al., 2021). Firstly, the viability assay was performed. Next, the parasite replication revealed that both compounds efficiently inhibited the *T. gondii* intracellular proliferation and triggered an irreversible effect on tachyzoites.

In agreement with our data, studies have already demonstrated that oleoresins from *Copaifera* present activity against some pathogens. *C. pubiflora* and its compound (*ent*-hardwickiic acid) showed antibacterial properties and should be explored as new therapeutic alternatives to treat oral diseases such as dental caries and endodontic infections (Moraes et al., 2020). Pieri (2012) determined that copalic acid and β-caryophyllene are common components obtained from oleoresins and suggested that the bioactivity of the *Copaifera* oleoresin on *Trypanosoma cruzi* replication is due to this synergism. Additionally, terpenes isolated from *Copaifera* oleoresins promoted changes in oxidative metabolism, induced autophagy and decreased the replicative forms of *T. cruzi*, in particular amastigotes (Izumi et al., 2012). Izumi et al. (2013) tested oleoresins of eight species of *Copaifera* against *T. cruzi*, and oleoresin from *C. martii* and *C. officinalis* exhibited the best activity against replicative forms of the parasite. A more recent study demonstrated that oleoresin and kaurenoic acid from *C. martii* decreased *T. cruzi* amastigote proliferation in murine peritoneal macrophages and HeLa cells (Kian et al., 2018). In addition, murine peritoneal macrophages treated with *C. reticulata* oil controlled significantly the infection by *Leishmania amazonensis* (Santos et al., 2008), while *C. martii* oil promoted a reduction on the size lesion caused by *L. amazonensis* in BALB/c (Santos et al., 2011). Similarly, the treatment with nanoemulsion from *Copaifera* led to a reduction in *L. infantum* and *L. amazonensis* infection levels in macrophage cultures, as well as presented beneficial effects on the lesion size and parasite burden in BALB/c (De Moraes et al., 2018). Also, human fibroblast treated with oleoresin from *C. reticulata* showed activity against the *Plasmodium falciparum* W2 and 3D7 strains and reduced the parasitemia in BALB/c (Souza et al., 2017). Finally, our previous study showed that oleoresins from *C. reticulata*, *C. duckei*, *C. paupera* and *C. pubiflora* induced a significant reduction on *T. gondii* infection in BeWo cells (Teixeira et al., 2020). Until the present moment, no study was conducted to verify the effects of hydroalcoholic extracts from *Copaifera* against *T. gondii*, systemically or in maternal-fetal interface; and also there is no study about the effect of the hydroalcoholic extract and oleoresin from *C. multijuga* on *T. gondii* infection. Thus, the present study is the first to show these effects.

It is important to emphasize that HTR8/SVneo cells are experimental model of human extravillous trophoblast cells (Souza et al., 2021). This type of trophoblast is important to invade the myometrium in order to interpose between the endothelial cells and guarantee the adequate blood supply to the embryo (Aplin et al., 2020). Our previous study demonstrated that extravillous trophoblasts are more susceptible to *T. gondii* infection in comparison to villous trophoblast (Oliveira et al., 2021). Thus, it is possible to conclude that extravillous trophoblast cells are a potential entry for tachyzoites gain the embryonic and placental tissues, maybe the major transmission route. In the present study, we verified that oleoresin and hydroalcoholic extract from *C. multijuga* reduced significantly the infection in HTR8/SVneo cells. It means that these compounds are great tools to control the vertical transmission of *T. gondii* when it occurs by the extravillous trophoblast route. One more time, the present study is the first to show these effects.

*T. gondii* is an obligate intracellular protozoan parasite capable of infecting a wide variety of nucleated cells (Dubey and Jones, 2008). Therefore, the successful establishment of the infection requires the ability of the parasite to adhere, invade and proliferate within the host cell (Hall et al., 2011; Adeyemi et al., 2017). Therefore, the next question that we would like to determine was the direct action of the hydroalcoholic extract and oleoresin from *C. multijuga* on tachyzoites. Pretreated parasite demonstrated a lower capacity to adhere, invade and proliferate in both cells. These findings are in agreement with our previous study, in which preincubated tachyzoites with oleoresins from *C. reticulata*, *C. duckei*, *C. paupera* and *C. pubiflora* showed lower rates of adhesion, invasion and proliferation in BeWo cells (Teixeira et al., 2020). However, other studies have already demonstrated that parasites pretreated with other drugs also showed reduced ability to infection. Castro-Filice et al. (2014) showed that the pretreatment of *T. gondii* with a combination of SDZ + PYR + folinic acid or azithromycin reduced the parasite proliferation after 72 h in human villous explants. Similarly, tachyzoites from atypical strains of *T. gondii* pretreated with azithromycin or spiramycin had a lower capacity to invade and replicate in BeWo cells (Ribeiro et al., 2017). Thus, the pretreatment with oleoresin and hydroalcoholic extract from *C. multijuga* suggested that these compounds can directly affect the parasite structure and, consequently, decrease its functional capabilities. A recent study declared that these natural compounds can affect different structures in *T. cruzi* and *Leishmania* spp, and it is hypothesized that the mitochondrion is a strategic target to induce parasite death (Lazarin-Bidóia et al., 2022). Also, *L. infantum* and *L. amazonensis* promastigotes treated with nanoemulsion from *Copaifera* presented ultrastructural alterations as oval cell shape and retracted flagella (De Moraes et al., 2018). Our previous study demonstrated that *C. paupera, C. reticulata, C. pubiflora* and *C. duckei* induced tethered *T. gondii* tachyzoites, which dampen the cytokinesis (Teixeira et al., 2020). Future studies are necessary to investigate the types of structural changes that occur in *T. gondii* when treated with these compounds from *C. multijuga.*

In a second moment, after demonstrating the effect of *C. multijuga* on tachyzoites replication, as well as adhesion and invasion, we investigated the immune response of host cells, suggesting that these compounds could be involved in the modulation of the host’s cellular environment.

It was observed that BeWo cells upregulated IL-6 and downmodulated IL-8. It is widely known that IL-6 is an important cytokine involved in *T. gondii* control in various cell types, such as macrophages and monocytes (Pereira et al., 2019) and human and murine trophoblasts (Barbosa et al., 2014; Barbosa et al., 2015; Gomes et al., 2018). Thus, it is plausible to suggest that the higher IL-6 in BeWo cells infected and treated with hydroalcoholic extract or oleoresin was important to control *T. gondii* replication. Although MIF is a cytokine important during *T. gondii* infection (Silva et al., 2017), the level of this cytokine increased only in uninfected treated-BeWo cells or oleoresin treated-cell at 32 µg/mL, then it seems that MIF did not contribute to parasite control when oleoresin or hydroalcoholic extract are used in BeWo cells. IL-8 has emerged as a cytokine important to disseminate *T. gondii* infection (Sommerville et al., 2013). Our previous study showed low levels of IL-8 in THP-1 cells infected by *T. gondii* and inhibited for cyclooxygenase 2 (COX-2), which contributes to the control of parasite replication (Pereira et al., 2019). In this sense, it is possible to hypothesize that the low levels of IL-8 in BeWo cells treated with the hydroalcoholic extract and oleoresin from *C. multijuga* could have contributed to *T. gondii* control. Future studies are necessary to verify the influence of *Copaifera* on COX-2 modulation. Therefore, in BeWo cells, the *T. gondii* control mediated by the hydroalcoholic extract and oleoresin from *C. multijuga* was an association between the direct action of these compounds on tachyzoites, as well as the immune response of these cells. For HTR8/SVneo cells, cytokines were slightly modulated (not statistically significant) when compared to untreated/infected cells. Thus, it is possible to conclude that cytokines did not contribute to *T. gondii* control in HTR8/SVneo cells when treated with compounds from *C. multijuga*. Thus, in this case, the direct action of these compounds on tachyzoites was the only mechanism to downmodulate infection in these cell types. As HTR8/SVneo cells are representative of human extravillous trophoblast (Souza et al., 2021), a potent route for congenital toxoplasmosis, and both oleoresin and hydroalcoholic extract controlled *T. gondii* infection in these cells without change significantly the cytokine profile, it is possible to conclude that both compounds from *C. multijuga* are an excellent alternative strategy to prevent vertical transmission and maintain the immune profile required for a successful pregnancy. Finally, the ROS production was not significant in infected and treated BeWo and HTR8/SVneo cells.

Finally, we investigated the effect of oleoresin and hydroalcoholic extract in human villous explants from third-trimester pregnancy, and we also observed reduced *T. gondii* intracellular proliferation, even with no change in cytokine profile, suggesting a direct effect of both compounds on tachyzoites. One more time, both compounds from *C. multijuga* showed to be an excellent alternative strategy to prevent vertical transmission and maintain the immune profile required for a successful pregnancy. Cell cultures are notorious examples of *in vitro* studies. However, the placental explants are very important to validate the data obtained with cell lineages, since these cells can be very different from the natural environment. Then, in studies of maternal-fetal interface, the association between *in vitro* (cells) and *ex vivo* (explants) studies (Rosini et al., 2022) is mandatory, and we demonstrated that both oleoresin and hydroalcoholic extract are enough to control *T. gondii* infection in both trophoblast cells and villous explants.

Based on the results of the study, the leaf hydroalcoholic extract and oleoresin obtained from *C. multijuga* presented different antiparasitic activities in a dependent manner of the experimental model. For BeWo, representative of human villous trophoblast, *C. multijuga* presented a direct action in tachyzoite and modulated the immune response, whereas only the direct effect in tachyzoites participated of the parasite control in HTR8/SVneo cells, representative of human extravillous trophoblast, and human explants. Considering all these parameters, the leaf hydroalcoholic extract and oleoresin from *C. multijuga* can be a target for the establishment of new therapeutic strategies for congenital toxoplasmosis. However, more detailed studies are needed to evaluate the bioactive components and other possible mechanisms of these compounds.

## Data availability statement

The original contributions presented in the study are included in the article/**Supplementary Material**. Further inquiries can be directed to the corresponding author.

## Ethics statement

The studies involving human participants were reviewed and approved by Ethics Committee of the Federal University of Uberlandia, MG, Brazil. Approval number 3.679.426. Written informed consent to participate in this study was provided by the participants’ legal guardian/next of kin.

## Author contributions

ST and BB design the experiments. AM, ST, GS, AR. JJ, GM, and BB performed the experiments. ST, KB, AM, and BB analyzed the data. SA, RV, JB, and CM were responsible for the identification and collection of *Copaifaera* spp. oleoresin and hydroalcoholic extract. BB, ST, AG, CM, and EF, participated in the data interpretation. AM and BB discussed the findings. ST, GS, and BB, reviewed the manuscript. All authors approved the final version of the manuscript.

## Supplementary material

Supplementary Figure 1 Cytokine production in villous explants. Explants were infected and treated or not with the hydroalcoholic extract or oleoresin from *C. multijuga* for 24 h. Untreated and uninfected explants (medium), untreated and infected explants (medium Tg) and SDZ + PYR were used as controls. Then, supernatants were collected and used to measure IL-6 **(A, B)**, IL-8 **(C, D)**, MIF **(E, F)** and TNF **(G, H)**. The results were expressed as means ± standard deviation of four experiments performed in six replicates. Significant differences detected by One-Way ANOVA, Bonferroni’s multiple comparisons post-test. ^*^ *P* < 0.05 in relation to medium (for uninfected cells). ^#^ *P* < 0.05 between medium and medium Tg.
