# Supplementary figures and images for: Leaf hydroalcoholic extract and oleoresin from Copaifera multijuga control Toxoplasma gondii infection in human trophoblast cells and placental explants from third-trimester pregnancy
Source: Front Cell Infect Microbiol. 2023 Feb 13;13:1113896. doi: 10.3389/fcimb.2023.1113896 (PMC9970041; doi:10.3389/fcimb.2023.1113896)

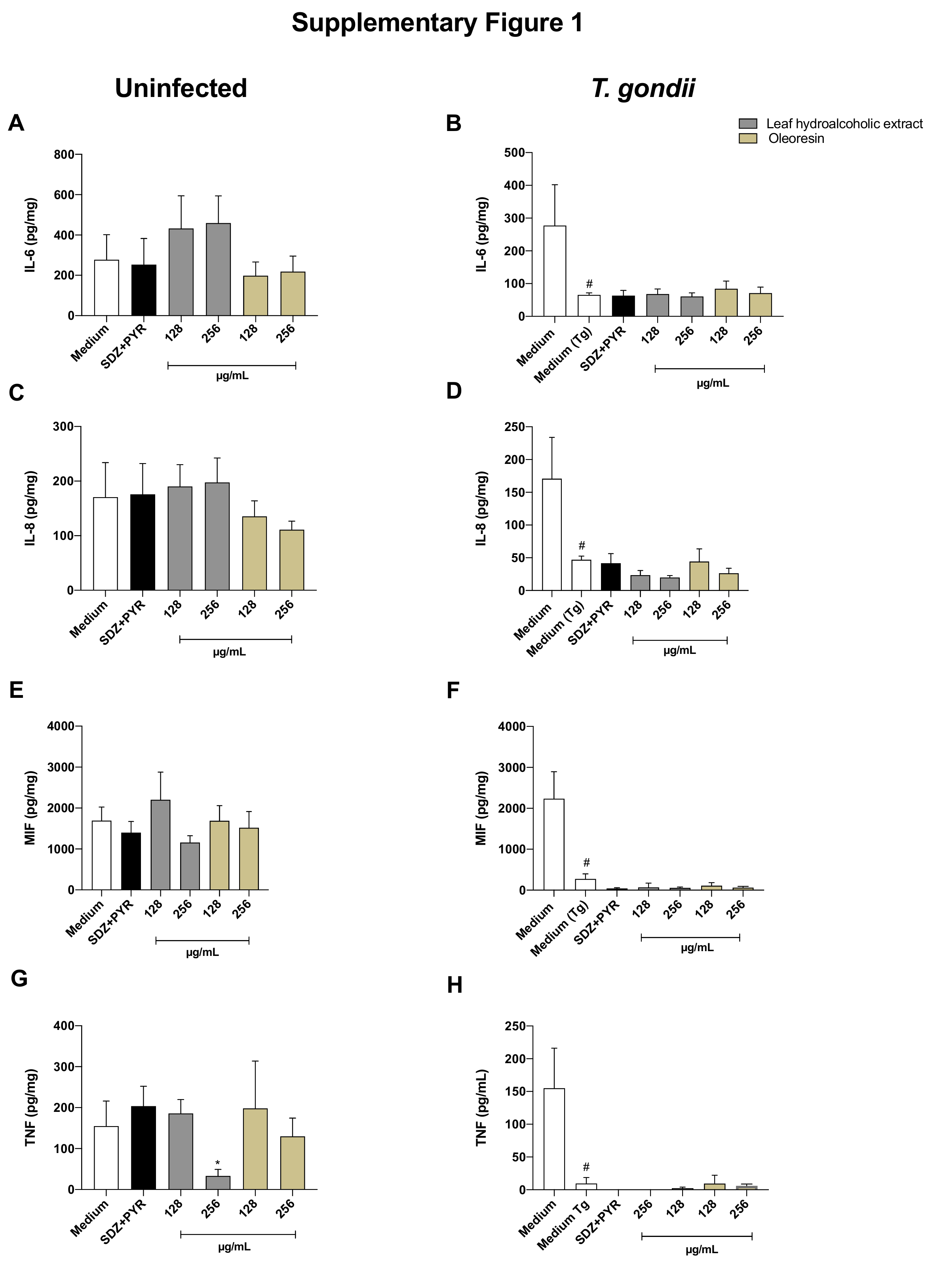

Supplement: Supplementary Figure 1 — Cytokine production in villous explants. Explants were infected and treated or not with the hydroalcoholic extract or oleoresin from C. multijuga for 24 h. Untreated and uninfected explants (medium), untreated and infected explants (medium Tg) and SDZ + PYR were used as controls. Then, supernatants were collected and used to measure IL-6 (A, B), IL-8 (C, D), MIF (E, F) and TNF (G, H). The results were expressed as means ± standard deviation of four experiments performed in six replicates. Significant differences detected by One-Way ANOVA, Bonferroni’s multiple comparisons post-test. * P < 0.05 in relation to medium (for uninfected cells). # P < 0.05 between medium and medium Tg. [file Image_1.tif]
